# Dietary Intake in Law Enforcement Personnel: Occupation Is an Additional Challenge for Changing Behavior

**DOI:** 10.3390/nu14071336

**Published:** 2022-03-23

**Authors:** Kristen L. MacKenzie-Shalders, Ka Wing Lee, Charlene Wright, Joe Dulla, Angela Tsoi, Robin M. Orr

**Affiliations:** 1 Nutrition and Dietetics, Faculty of Health Sciences and Medicine, Bond University, Robina 4226, Australia; kmackenz@bond.edu.au (K.L.M.-S.); kawing.lee@student.bond.edu.au (K.W.L.); cwright@bond.edu.au (C.W.); angela.tsoi@student.bond.edu.au (A.T.); 2 School of Medicine and Dentistry, Centre of Applied Health Economics, Menzies Health Institute Queensland, Griffith University, Southport 4215, Australia; 3 Tactical Research Unit, Bond University, Robina 4226, Australia; jdulla@sandiego.edu

**Keywords:** police, food choice, behavior, nutrition, health, framework

## Abstract

Background: Law enforcement is a dangerous, stressful, and health-threatening occupation. This study investigated the association between demographic factors including sex, age, and types of law enforcement occupation and described self-reported barriers to healthy and enjoyable diets within a cohort of law enforcement personnel. Methods: This mixed-methods study explored cross-sectional data from the Perceived Barriers to Healthy Eating validated survey. The survey included eight questions obtaining quantitative data and two open ended question obtaining qualitative data. A framework thematic analysis using the theory of planned behavior was undertaken to describe self-reported barriers to a healthy and enjoyable dietary intake. Results: 159 participants (median age = 27 (range 19–60) years; 74% males) were surveyed. In general, demographic factors are not associated with the dietary beliefs and behavior of law enforcement personnel. Self-reported barriers (generated themes) to a healthy and enjoyable diet included individual preferences, social influences, internal drive, capacity to change and occupational influences, which broadly aligned with the theory of planned behavior. Conclusions: Dietary intake in law enforcement personnel is impacted by occupational considerations, including busy schedules, long working hours, inconsistent meal breaks, tiredness, and shift work. The study provides useful information to support dietary interventions.

## 1. Introduction

Law enforcement is considered to be a dangerous, stressful, and health-threatening occupation [1]. Law enforcement personnel are required to respond to a variety of physically and mentally challenging tasks and, while their occupation can be sedentary in nature, this is interspersed with high-intensity activity [1]. As such, law enforcement personnel require a requisite level of fitness in order to perform these tasks safely. In addition, general health and wellbeing is of importance. Police officers are known to be at an increased risk of cardiovascular disease (CVD) when compared to the general public [2,3,4,5]. Furthermore, law enforcement as a profession is known to have high levels of hypertension, cholesterol, and obesity [2,6]; all of which are risk factors for CVD [6]. Potential reasons for poor officer health, identified in the Safety and Health Improvement: Enhancing Law Enforcement Departments (SHIELD) study, include high stress, sleep deprivation, rotating work shifts, overtime duties, and unhealthy lifestyles (inclusive of poor diet) [7,8].

Given that diet contributes both to physical performance and health, it is postulated to play a role in law enforcement capability. A recent review of the free-living dietary intake of tactical personnel, inclusive of military, fire and rescue and law enforcement, found that persons serving in these occupations failed to meet energy and carbohydrate recommendations, met dietary protein recommendations and exceeded dietary fat recommendations [9]. While dietary under-reporting is a consideration in these populations [10], studies have reported that officers follow high fat and energy dense diets with a high dietary intake of processed foods and drinks [11,12,13], which were noted as affecting their health status [11]. 

These findings are not surprising given that officers are often required to work in shifts and that shift work, defined as a working period that differs from conventional working hours [14], is known to impact circadian rhythms, hunger and dietary outcomes [15,16,17]. A recent preceding study in the same cohort, using the perceived barriers to health eating questionnaire [18,19,20], also described several key barriers to dietary intake in this population including busy lifestyle and irregular working hours [21]. The study postulated that, rather than a lack of dietary knowledge and willingness to change, barriers to the implementation of healthy eating may present as key limitations in law enforcement personnel. Thus, interventions should focus on behavior change strategies which address reported barriers to healthy eating (the ‘how’ of change) [21]. 

However, the perceived barrier to healthy eating questionnaire is validated in civilians and therefore the open-ended questions (i.e., where the questionnaire allows law enforcement personnel to self-report barriers to dietary intake) may provide additional information. Of note in health research, the theory of planned behavior is commonly used to examine attitudes and behaviors [22]. Behavioral control plays a central role in the theory’s current formulation, and the degree of actual control moderates the effect of intention on behavior. Addressing barriers (i.e., factors that may prevent individuals from acting on their intentions) may be central to dietary interventions in law enforcement personnel, particularly as they have described a willingness to change [21]. 

Noting that healthy dietary and good nutritional practices are essential for law enforcement personnel to optimize their physical performance [10,15] and health [23], and that barriers to healthy eating could be a leading factor in poor dietary patterns [21], identifying barriers to healthy eating for law enforcement populations is of importance. In addition, studies have shown preliminary differences and/or requested further research in the exploration of differences in dietary intake patterns due to age [10], sex [24] and patterns across industries (occupations) and shift-types [17]. A recent systematic literature review demonstrated inconsistent reporting of these variables [9] and, due to these plausibly impacting barriers to dietary intake, further exploration is warranted. 

Thus, this mixed-methods study aimed to (1) investigate the association between demographic factors such as sex, age, and types of law enforcement occupations and self-reported barriers to achieving a healthy diet and (2) investigate the self-reported barriers to healthy eating of law enforcement personnel, including during academy training and general duties, by using a framework thematic analysis. It is crucial to understand these barriers if targeted and specific interventions are to be effectively implemented in the future.

## 2. Materials and Methods

### 2.1. Study Design 

This study had a mixed-methods design, with qualitative and quantitative data obtained from surveys, and had two objectives. The first objective, previously reported [21], was to investigate the dietary habits and influencing factors or barriers in law enforcement personnel. This focused on the quantitative results from three validated surveys including the Perceived Barriers to Healthy Eating, Food Choice Questionnaire, and Rapid Eating Assessment for Participants, Short Version [18,19,20]. The current study focuses on the second objective which was to determine any associations between sex, age, and type of role with the responses to the Perceived Barriers to Healthy Eating questionnaire. In addition, this study aimed to investigate the qualitative data obtained from the questionnaire regarding self-reported barriers using a qualitative framework analysis which involved mapping data to the theory of planned behavior. Ethics approval was provided by the International Review Board (IRB 15-074) and Bond University’s Human Research Ethics Committee (RO1927). This study is reported in accord with the STROBE and COREQ guidelines [25,26].

### 2.2. Sample 

Participants were recruited from a law enforcement agency in the United States of America. Sworn and non-sworn (i.e., under training) law enforcement officers aged ≥18 years were eligible for recruitment. Personnel were recruited from one US law enforcement agency alongside their training as part of their usual occupation. All participants provided informed consent prior to involvement in the study [21]. 

### 2.3. Data Collection 

The paper-based surveys (written in English only) were distributed to the recruited participants and included demographic questions and validated surveys. The Perceived Barriers to Healthy Eating questionnaire contains five questions with a statement in which participants are required to state if they agree, disagree or neither agree nor disagree, for example, “I would like to change what I eat”. One question with a 5-point Likert scale asked, “How healthy do you think you have been eating in the past 12 months?” with one being very unhealthy and five being very healthy. Another question provided a list of 22 possible barriers for participants to select which they perceive as major difficulties in trying to eat a healthier diet and the final quantitative question provides 10 possible options and asks participants to select the three main options that mostly apply to them regarding “What healthy habits could you improve on?”, for example, “Eat more fruits”. The final questions provide the opportunity for qualitative responses to the questions “Please state your biggest challenge/barriers to eating a healthy and enjoyable food intake” and “Do you have any additional comments?” [21]. All data were collected, de-identified, and entered into Microsoft Excel (version 365, Microsoft) for management. All data were stored securely on servers at Bond University and only project researchers had access to the data. Qualitative responses were typed verbatim.

### 2.4. Data Analysis 

#### 2.4.1. Quantitative Data 

The results of the Perceived Barriers to Healthy Eating questionnaire have been previously reported [21]. Data analysis in the current study focused on associations between sex, age, and type of role with the questionnaire responses. Data were analyzed using the Statistical Package for the Social Sciences (SPSS) software (v. 26, IBM). The variable of age was categorized into five groups (aged 19–24, 25–29, 30–34, 35–39 and ≥40 years), based on previous law enforcement studies [27]. Categorical variables were reported as frequency (*n*) and total percentage with all percentages referring to the valid data available for the variable. Chi-square tests were used to report associations, or Fisher’s exact tests (two-sided) were used when the results had ≥20% of cells with an expected count of less than five. Alpha levels were set at *p* < 0.05 a priori. For two of the questions participants could select multiple responses; therefore, the significant differences could not be retrieved.

#### 2.4.2. Qualitative Data 

For the qualitative data obtained, framework analysis was conducted [28]. The procedure for analysis first included transcribing handwritten survey responses word-for-word, becoming familiar with the responses, followed by coding [22,28]; three authors (KL, KMS & AT) were responsible for data coding, one author consolidated all the coding (KL) and one author (KMS) was responsible for refining the codes. The data were coded according to the nature of the response, categorized, and grouped into themes. The themes were developed by two authors (KL & KMS) by referring to the established codes. The theory of planned behavior was applied and data was mapped to the framework [22]. The theory of planned behavior is commonly used to examine attitudes and behaviors [29]. It proposes that intentions influence behavior and that intentions are influenced by attitude, subjective norm, and perceived behavioral control [29]. The theory was used to explain the reported behaviors and barriers to healthy eating in the cohort and identify any differences in these reported behaviors and barriers to those applying in the general population.

## 3. Results

There were 159 participants (median age = 27 (range 19–60) years; 74% males) recruited from a law enforcement agency in the United States of America. The participants were employed as custody assistants or civilian jailers (*n* = 40), reserve peace officers (*n* = 20) and sworn deputy and police officer recruit academy trainees (*n* = 99). For this study, the terms trainee and recruit are equivalent. All participants were enrolled in their respective initial occupational training academies, which differed in length of training due to occupational, state, and organizational requirements. Thus, all participants participated as trainees/recruits undergoing initial occupational training as opposed to personnel serving in incumbent roles.

### 3.1. The Association between Sex, Age, and Type of Occupation with Behavior and Willingness to Achieve a Healthier Diet

It was found that there was no significant difference in sex, age, and type of occupation for all survey questions except for one (Table 1). Significant differences were seen for the question “How healthy do you think you have been eating in the past 12 months?” for age (*p* = 0.029) and types of occupation (*p* = 0.040) (Table 2). A higher proportion of the age group 25–29 reported ‘somewhat healthy’ eating in comparison to the other age groups Additionally, a higher proportion of sworn deputy and police officer trainees responded as “Somewhat healthy” in comparison to custody assistants or civilian jailers and reserve peace officers, as outlined in Table 2. 

### 3.2. Self-Reported Barriers to Achieving a Healthier and More Enjoyable Food Intake

As a result of thematic analysis to the question ‘Please state you biggest challenge/barriers to eating a healthy and enjoyable food intake’, four main themes were identified. These included ‘Individual preferences’, ‘Social influences’, ‘Internal drive and capacity to change’ and ‘Occupational considerations’, as seen in Table 3. 

### 3.3. Mapping to the Theory of Planned Behavior

**Figure 1:**
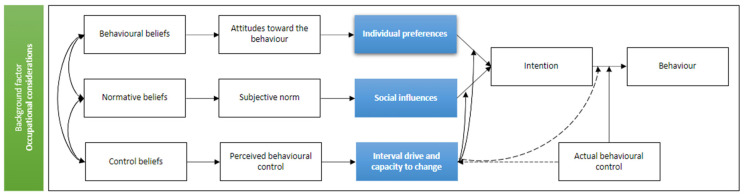
Themes mapped to the theory of planned behavior (Adapted from Ajzen 2019) [30].

Three of the four themes aligned with the different components in the Theory of planned behaviour, which can be seen in Figure 1 and Table 3.

## 4. Discussion

This mixed-methods study aimed to investigate the association between demographic factors such as sex, age, and types of law enforcement occupations and self-reported barriers to achieving a healthy diet. It also aimed to investigate the self-reported barriers to healthy and enjoyable eating in law enforcement personnel, including during academy training and general duties, by using a qualitative framework analysis. 

The findings of this study suggest that demographic factors are not associated with the dietary beliefs and behaviors of law enforcement personnel. Law enforcement personnel in the study cohort faced similar challenges to consuming a healthy diet independent of their occupation, age, or sex. Only minor differences existed between age group 25–29 years and sworn deputy and police officer trainees and other groups, who reported eating ‘slightly healthy’ in comparison to ‘neutral’ for the previous 12 months. 

Further, while law enforcement personnel are willing to make changes and improve their diet [21], several barriers were identified that prevent them from achieving a healthy and enjoyable diet. Primary barriers (themes) included individual preferences, social influences, occupational influences, and internal drive and capacity to change. The themes identified broadly aligned with the theory of planned behavior framework as outlined in Figure 1 and are explored further below.

### 4.1. Individual Preferences 

Personal food preferences were reported as a common barrier to maintaining a healthy diet. For example, a preference for sweet foods, specific foods or food groups, hot/warm foods and a variety of foods were reported. These hedonistic desires have been recently described in the scholarly literature and may partially relate to human consumption being driven by pleasure, and not just the need for energy [31]. A recent systematic review supported that meal timing and food choice at night (e.g., high intake of less healthy or sweet snack foods), rather than daily energy intake per se, is likely to impact dietary intake in shift workers [13].

Interestingly, while food preferences were reported by participants to be a barrier to healthy eating, this overlooked that the questionnaire specifically asked for barriers to healthy and *enjoyable* eating. What constitutes a ‘healthy’ diet is open to interpretation, and preferences or cultural acceptability can reasonably form one part of its definition. Participants felt that healthy eating was less enjoyable, known as an experiential behavioral belief [28]. The scholarly literature has also supported that the ‘unhealthy = tasty intuition’ suggests that healthy foods and diets are often perceived as ‘less palatable, tasty or enjoyable [32].’ Behavioral beliefs are theorized to produce a positive or negative attitude toward behaviors, and current experiential behavioral beliefs may be producing negative attitudes in this population. For tactical personnel, ensuring that they have access to, and learn how to source and prepare, healthy and tasty and/or enjoyable food is a common-sense approach to supporting positive dietary change. 

### 4.2. Social Influences

Primary reported social influences included families, partners, and children, which impacted the types of food and meals consumed by law enforcement personnel. Co-workers were also commonly reported to influence food and fluid choices, and peer pressure was reported. Several studies have found that healthy behaviors are influenced by the social group and the workplace environment, where there is a collegial influence in the selection of food, food preparation and quantity consumed [13,33,34], which further supports the importance of interventions not just supporting the individual, but also the workplace environment and culture. There have been some reported, positive shifts in workplace culture regarding healthy eating [13], but individual workplaces will need to consider this component when undertaking interventions or support to enhance dietary intake.

This theme aligns with the constructs of normative beliefs and subjective norms in the theory of planned behavior, and similarly the examples highlighted recognised the importance of both ‘descriptive’ and ‘injunctive’ components, i.e., the expectation that someone else approves or disapproves of a certain behavior (injunctive) and the modelling of others performing the behavior (descriptive) [22]. 

### 4.3. Internal Drive and Capacity to Change

The internal drive of law enforcement personnel was also reported as a key factor influencing dietary intake. A previously published study from the same participants demonstrated that law enforcement personnel are willing to change dietary habits [21], a finding which has also been shown in other tactical personnel [13]. However, a range of factors impact their internal drive and capacity to change. For example, participants reported tiredness and a lack of energy and willpower, most likely at point-of-consumption, as a barrier to dietary intake. Therefore, while dietary intake undoubtedly impacts health directly, a less healthy dietary intake in tactical personnel is also a symptom of broader non-health supporting behaviors, such as a lack of sleep due to shift work. 

Similarly, this disjoint between motivation (intention) and behavior aligns with the theory of planned behavior, which also emphasizes that intention, does not necessarily translate to the desired behavior, due to the actual behavioral control of the individual over the desired behavior—in this case healthy and enjoyable eating [22]. Within this study, one of the factors that is likely to have exacerbated this issue was the individuals’ self-reported capacity to change, which included examples such as a lack of knowledge of (healthy) food and snacks, how to prepare healthier meals and snacks, or to access healthier foods and food ingredients. In addition, while the participants reported that they had been previously motivated to cook healthy meals, due to several experiences of failure this had been discontinued. 

### 4.4. Occupational Considerations

Law enforcement personnel reported irregular work patterns, inconsistent meal breaks, long working hours, shift work, and busy working schedules, all contributing as barriers to healthy eating. Describing an occupational component, independent of other factors that influence dietary choice in tactical personnel, is not new [13,35,36]. These factors were also reported to have contributed to snacking behavior, poor food choices, and reliance on convenient fast-food and high energy snacks, as has been described elsewhere [12,13]. 

A previous mixed-methods study in fire and rescue personnel also found two primary themes impacting dietary intake; (1) shift schedules influence the type of food and snacks at work and (2) food choices during a shift are dependent on time availability and ease of access. For example, take-away food (commonly known as “fast or convenience food”) choice was reported as being dependent on what was available at the time and in the location [13]. In addition, the availability of less healthy food options at stations, and in the work culture have also been described as influencing dietary intake in tactical personnel [13,37]. A study in Australian paramedics also strongly supported the occupational/organizational domain as a critical factor impacting dietary choice, and thus as a point-of-intervention for tactical personnel. These personnel, similar to some law enforcement personnel, can often be based out of a vehicle for a shift which has been shown to influence dietary intake [36,38]. 

For this and the other identified themes, there were interactions between the themes. For example, participants reported food costs or perceived expense of healthy foods as a barrier to healthy intake and thus their capacity to change, which was also linked to their income from their occupation. 

### 4.5. Implication for Future Practice and Research

This study did not find major differences between demographic factors such as sex, age, and types of law enforcement occupations and dietary beliefs and barriers, and therefore, considering the similarity within this population, a behavioral change intervention aimed at the entire law enforcement agency may be feasible. The qualitative component of this study provided formative research in identifying key predictors of decision making for healthy and enjoyable eating practices. 

Dietary interventions and health promotion programmes at the workplace have been shown to benefit the wellbeing and dietary behaviors of shift workers through education and a supportive environment [23,39]. In addition, interventions that support a positive culture and environment have been shown to promote positive health behaviors [33]. As such, this study has supported behavior change interventions informed by the theory of planned behavior [22] and address the following key areas.

- Supporting health promoting food choices that are also enjoyable/meet individuals food preferences e.g., supporting personnel to recognise and enjoy healthier take-away options.
- Recognize, support, and address the role of family, friends, and peers in positive eating behaviors, e.g., cooking or shopping interventions that include family members and those that support positive food culture in the agency.
- Recognize individuals’ motivation to change and discuss strategies to overcome common barriers as described within this study and its precursor study [21], e.g., busy lifestyle and irregular working hours.
- Explore and address opportunities for interventions within the person’s occupation, e.g., food intake provision or messaging within the workplace/station.

Where occupational interventions are conducted, identifying, assessing, and reporting desired outcomes (particularly in scholarly literature) would be beneficial to informing practice.

For future studies, applicability of this approach to other tactical personnel (e.g., fire and rescue, paramedics) or law enforcement agencies from other countries may be beneficial. Interestingly, a study in paramedics described several themes impacting paramedics’ food choices; these were physiological (hunger and cravings, fatigue and physical health), physical (availability and access, station location, convenience and ambulance environment), organizational (shift work, workload and meal break structure) and psychosocial domains (lifestyle choices, experience, and colleagues) [36] which align with the current study. In addition, studies that support the development of new dietary guidelines and support the efficacy of interventions would be beneficial. 

### 4.6. Strength and Limitations

This mixed-methods study consisted of qualitative and quantitative data analysis, which provided a better understanding of the barriers to healthy and enjoyable eating among the law enforcement population. It clearly identified an occupational element as a barrier to a healthy and enjoyable diet in law enforcement personnel, alongside other themes that mapped to the theory of planned behavior. The study broadly covered a range of law enforcement personnel in different vocations undergoing initial academy training, including custody assistants, reserve peace officers, and sworn deputy and police officer trainees. However, it was limited to one cohort of law enforcement personnel in the United States of America and as such should be interpreted for its translatability to other occupations and countries. Considering the exploratory nature of the project, the extremely diverse nature of US law enforcement training standards, delivery, and confidentiality required, this survey delivery method was appropriate. While the sample size is not large, it is reasonable considering the difficulties in engaging tactical personnel in dietary research due to their competing commitments and priorities. 

## 5. Conclusions

Law enforcement personnel are willing to change their dietary intake, but implementing healthy behaviors are impacted by barriers including their individual preferences (including for less healthy foods that are perceived as tastier), social influences (friends, peers, and family), internal drive and capacity to change (knowledge, skill, willpower, and tiredness), and occupational influences (busy schedules, long working hours, inconsistent meal breaks, and shift work). For the study sample, demographic factors were generally not associated with dietary beliefs and behaviors. The study findings are useful in informing future occupational interventions to improve the dietary behaviors of law enforcement personnel.

## Figures and Tables

**Table 1:** The association between sex, age, and type of occupation and the dietary beliefs and behaviors of law enforcement personnel.

Category	Agree	Disagree	Neither Agree or Disagree	*p*-Value
I would like to change what I eat				
Male	49 (42%)	16 (14%)	51 (44%)	0.576
Female	18 (45%)	3 (8%)	19 (48%)	
19–24 years	23 (43%)	8 (15%)	23 (43%)	0.786
25–29 years	20 (43%)	8 (17%)	19 (40%)	
30–34 years	11 (55%)	2 (10%)	7 (35%)	
35–39 years	6 (33%)	1 (6%)	11 (61%)	
≥40 years	8 (40%)	2 (10%)	10 (50%)	
Custody assistants or civilian jailers	22 (55%)	3 (8%)	15 (38%)	0.240
Reserve peace officers	12 (60%)	1 (5%)	7 (35%)	
Sworn deputy and police officers	34 (34%)	17 (17%)	48 (49%)	
We/I usually buy the same foods each week				
Male	86 (74%)	21 (18%)	9 (8%)	0.663
Female	28 (70%)	7 (18%)	5 (13%)	
19–24 years	40 (74%)	9 (17%)	5 (9%)	0.501
25–29 years	32 (68%)	8 (17%)	7 (15%)	
30–34 years	14 (70%)	4 (20%)	2 (10%)	
35–39 years	16 (89%)	2 (11%)	0 (0%)	
≥40 years	15 (75%)	5 (25%)	0 (0%)	
Custody assistant or civilian jailers	26 (65%)	8 (20%)	6 (15%)	0.240
Reserve peace officers	18 (90%)	2 (10%)	0 (0%)	
Sworn deputy and police officers	73 (74%)	18 (18%)	8 (8%)	
Healthy foods cost more				
Male	82 (71%)	18 (16%)	16 (14%)	0.615
Female	27 (68%)	5 (13%)	8 (20%)	
19–24 years	39 (72%)	10 (19%)	5 (9%)	0.684
25–29 years	32 (68%)	7 (15%)	9 (17%)	
30–34 years	13 (65%)	4 (20%)	3 (15%)	
35–39 years	13 (72%)	2 (11%)	3 (17%)	
≥40 years	12 (60%)	2 (10%)	6 (30%)	
Custody assistant or civilian jailers	30 (75%)	4 (10%)	6 (15%)	0.518
Reserve peace officers	11 (55%)	4 (20%)	5 (25%)	
Sworn deputy and police officers	68 (69%)	17 (17%)	14 (14%)	
I like to try new foods				
Male	91 (80%)	9 (8%)	14 (12%)	0.672
Female	33 (83%)	4 (10%)	3 (8%)	
19–24 years	42 (78%)	5 (9%)	7 (13%)	0.310
25–29 years	41 (89%)	3 (7%)	2 (4%)	
30–34 years	18 (90%)	1 (5%)	1 (5%)	
35–39 years	10 (59%)	3 (18%)	4 (24%)	
≥40 years	15 (75%)	2 (10%)	3 (15%)	
Custody assistant or civilian jailers	30 (79%)	3 (8%)	5 (13%)	0.388
Reserve peace officers	13 (65%)	3 (15%)	4 (20%)	
Sworn deputy and police officers	83 (84%)	8 (8%)	8 (8%)	
I am influenced by other family members/peers in my choice about what I eat				
Male	35 (30%)	59 (51%)	21 (18%)	0.217
Female	16 (40%)	21 (53%)	3 (8%)	
19–24 years	23 (43%)	24 (44%)	7 (13%)	0.364
25–29 years	14 (30%)	26 (57%)	6 (13%)	
30–34 years	3 (15%)	12 (60%)	5 (25%)	
35–39 years	4 (22%)	10 (56%)	4 (22%)	
≥40 years	9 (45%)	9 (45%)	2 (10%)	
Custody assistant or civilian jailers	16 (41%)	14 (36%)	9 (23%)	0.054
Reserve peace officers	10 (50%)	8 (40%)	2 (10%)	
Sworn deputy and police officers	27 (27%)	59 (60%)	13 (13%)	

**Table 2:** The association between sex, age, and type of occupation and participants’ response to “How healthy do you think you have been eating in the past 12 months?”.

Category	Very Unhealthy	Somewhat Unhealthy	Neutral	Somewhat Healthy	Very Healthy	*p*-Value
How healthy do you think you have been eating in the past 12 months?						
Male	0 (0%)	8 (7%)	52 (45%)	47 (41%)	9 (8%)	0.386
Female	0 (0%)	1 (3%)	23 (58%)	12 (30%)	4 (10%)	
19–24 years	0 (0%)	4 (7%)	29 (54%)	20 (37%)	1 (2%)	0.029 *
25–29 years	0 (0%)	2 (4%)	20 (43%)	21 (45%)	4 (9%)	
30–34 years	0 (0%)	0 (0%)	9 (45%)	9 (45%)	2 (10%)	
35–39 years	0 (0%)	0 (0%)	7 (39%)	6 (33%)	5 (28%)	
≥40 years	0 (0%)	4 (20%)	10 (50%)	4 (20%)	2 (10%)	
Custody assistant or civilian jailers	0 (0%)	2 (5%)	22 (55%)	12 (30%)	4 (10%)	0.040 *
Reserve peace officers	0 (0%)	4 (20%)	11 (55%)	3 (15%)	2 (10%)	
Sworn deputy and police officers	0 (0%)	4 (4%)	42 (42%)	45 (46%)	8 (8%)	

* Significant *p* ≤ 0.05.

**Table 3:** Themes and representative quotes.

Generate Theme and Representative Quotes	Mapped Theory of Planned Behavior Construct
**Individual preferences**	**Behavioral beliefs and attitudes towards the behavior**
Personal food and taste preferences were reported as a common barrier to a healthy and enjoyable food intake. For example, a preference for sweet foods, specific foods or food groups, hot/warm foods and specific food varieties were reported. *“The biggest challenge is to give up the foods I like, for example eating poultry and meat’—Male, 45, Sworn deputy or police officer* *“Too much sugary sweets when I go to the store that looks better than healthy foods”—Male, 23, Custody assistant or civilian jailer*	A behavioral belief is an individual’s expectation that behavior leads to certain outcomes (instrumental behavioral belief) or involves certain experiences (experiential behavioral beliefs). For example, the belief that eating health (the behavior) improves heart heath (the outcome) or that it is enjoyable (the experience). Behavioral beliefs are thought to produce a positive or negative attitude toward the behavior [22].
**Social influences**	**Normative beliefs and subjective norm**
The food preferences of family, partners, friends and peers were reported as barriers which influence participants’ food intake, especially if family and partners were involved in preparing meals. Additionally, a lack of social support and peer pressure were reported as barriers, including when participants were attempting to improve their dietary intake. *“I meal prep and eat healthy most of the time. I usually get made fun of because I ‘eat healthy’.”—Female, 30, Sworn deputy or police officer* *“I cook my own lunches, but my mom always prepares dinner and I eat food that is high in carbs, cholesterol (fat) (SIC) with no nutrients.”—Female, 28, Reserve peace officer*	There are two types of normative beliefs: injunctive and descriptive. An injunctive belief is the expectation that individuals such as friends, family, spouse, or co-workers approve or disapprove of performing certain behaviors. Descriptive bdliefs are beliefs as to whether important others themselves perform the behavior. Both contribute to the overall perceived social pressure to engage in the behavior or subjective norm [22].
**Internal drive and capacity to change**	**Control beliefs and perceived behavioral control**
Participants internal drive, particularly willpower, tiredness, and lack of motivation, were reported as barriers to a healthy and enjoyable food intake. In addition, participants described factors such as emotions and knowledge as influencing their food intake. Many participants reported a lack of food knowledge and cooking skills, knowledge of portion sizes and healthy food ideas. *“Willpower, after a long day at work it’s easier to pick up food rather than preparing a meal at home”—Male, 35, Reserve peace officer* *“My biggest challenge is probably my cooking skills. I generally stick to preparing a few healthy dishes I’m familiar with. Expanding on those skills would help me not get sick of having the same food all the time. Time is also a slight issue. It takes more willpower to meal prep when you’re busy.”—Female, 25, Sworn deputy or police officer*	Control beliefs concern the presence of factors that can either facilitate or impede performance of behaviors. Examples of control factors include skill, availability of time, money and so forth. A control belief is defined by an individual’s expectation that these factors will be present when they are trying to enact a particular behavior [22].
**Occupational considerations**	**Background factors**
Participants reported that the nature of their occupation posed a barrier to achieving a healthy and enjoyable food intake. Commonly reported barriers included busy schedules, long working hours, inconsistent meal breaks, tiredness, and shift work. Some participants also described money and a perceived higher cost of healthy foods as barriers. *“I like cook(ed) vegetables. If I bought cooked vegetables for lunch I wouldn’t be able to heat them up”—Male, 34. Custody assistant or civilian jailer* *”Due to long work hours, the time for healthy food preparation becomes a challenge”—Male, 34, Reserve peace officer*	Many factors not included in the theory of planned behavior may influence intentions and behavior. These may include demographic characteristics, personality traits, life values and so forth. These variables are considered background factors that have no direct effect on behavior, but can influence it indirectly [22].

## Data Availability

Data can be requested from the research team.
